# Predicting Maximum Tree Heights and Other Traits from Allometric Scaling and Resource Limitations

**DOI:** 10.1371/journal.pone.0020551

**Published:** 2011-06-13

**Authors:** Christopher P. Kempes, Geoffrey B. West, Kelly Crowell, Michelle Girvan

**Affiliations:** 1 Department of Earth Atmosphere and Planetary Sciences, Massachusetts Institute of Technology, Cambridge, Massachusetts, United States of America; 2 The Santa Fe Institute, Santa Fe, New Mexico, United States of America; 3 Portage, Los Alamos, New Mexico, United States of America; 4 Department of Physics and the Institute for Physical Science and Technology, University of Maryland, College Park, Maryland, United States of America; 5 Institute for Advanced Study, Princeton, New Jersey, United States of America; University of Hull, United Kingdom

## Abstract

Terrestrial vegetation plays a central role in regulating the carbon and water cycles, and adjusting planetary albedo. As such, a clear understanding and accurate characterization of vegetation dynamics is critical to understanding and modeling the broader climate system. Maximum tree height is an important feature of forest vegetation because it is directly related to the overall scale of many ecological and environmental quantities and is an important indicator for understanding several properties of plant communities, including total standing biomass and resource use. We present a model that predicts local maximal tree height across the entire continental United States, in good agreement with data. The model combines scaling laws, which encode the average, base-line behavior of many tree characteristics, with energy budgets constrained by local resource limitations, such as precipitation, temperature and solar radiation. In addition to predicting maximum tree height in an environment, our framework can be extended to predict how other tree traits, such as stomatal density, depend on these resource constraints. Furthermore, it offers predictions for the relationship between height and whole canopy albedo, which is important for understanding the Earth's radiative budget, a critical component of the climate system. Because our model focuses on dominant features, which are represented by a small set of mechanisms, it can be easily integrated into more complicated ecological or climate models.

## Introduction

A critical component for understanding the earth system is determining the interplay between biotic and abiotic factors, such as the interaction between forest characteristics and local meteorology [1]–[11]. At present a range of ecological perspectives and techniques are used for interpreting forest structure and dynamics at both the local and regional scale. Historical and ongoing modeling efforts have become increasingly accurate at describing critical forest features such as standing biomass and dynamic transpiration rates [4], [5], [11]–[20]. Most of these models explicitly simulate the temporal and/or spatial dynamics of a forest and typically focus on a detailed description of a variety of coupled plant processes including transpiration, competition between trees, seedling dispersal, and mortality.

Another perspective for interpreting ecological features is the use of allometric relationships as a means to characterize the general variation of plant traits across many species living in a variety of environments [21]–[24]. These scaling relationships show that, on the average, many of the dominant physiological traits relevant to forest dynamics and structure are correlated with tree size following approximate power laws (e.g. [22], [25]–[27]). As such, size is viewed as the major determinant of variation among trees setting the baseline from which variation due to local, environmental, historical, geographical, and species related factors are considered secondary perturbations. Because of the relative simplicity of these relationships many models rely on basic allometries as part of a more complicated description of plant behavior (e.g. [12], [19]). Furthermore, there are conceptual frameworks from which these scaling laws, at both the individual and community level, have been derived (e.g. [21], [28]–[30]). On the other hand there is ongoing debate over the exact value of the empirical exponents of each relationship and the range of tree sizes over which they are valid, and, in general, it is not yet known what sets the dominant variability of the data around a given scaling law (see [24] for a review). Thus, it is unclear how useful the basic power-law relationships are in describing local variation or how applicable they are to modeling endeavors.

**Figure 1:**
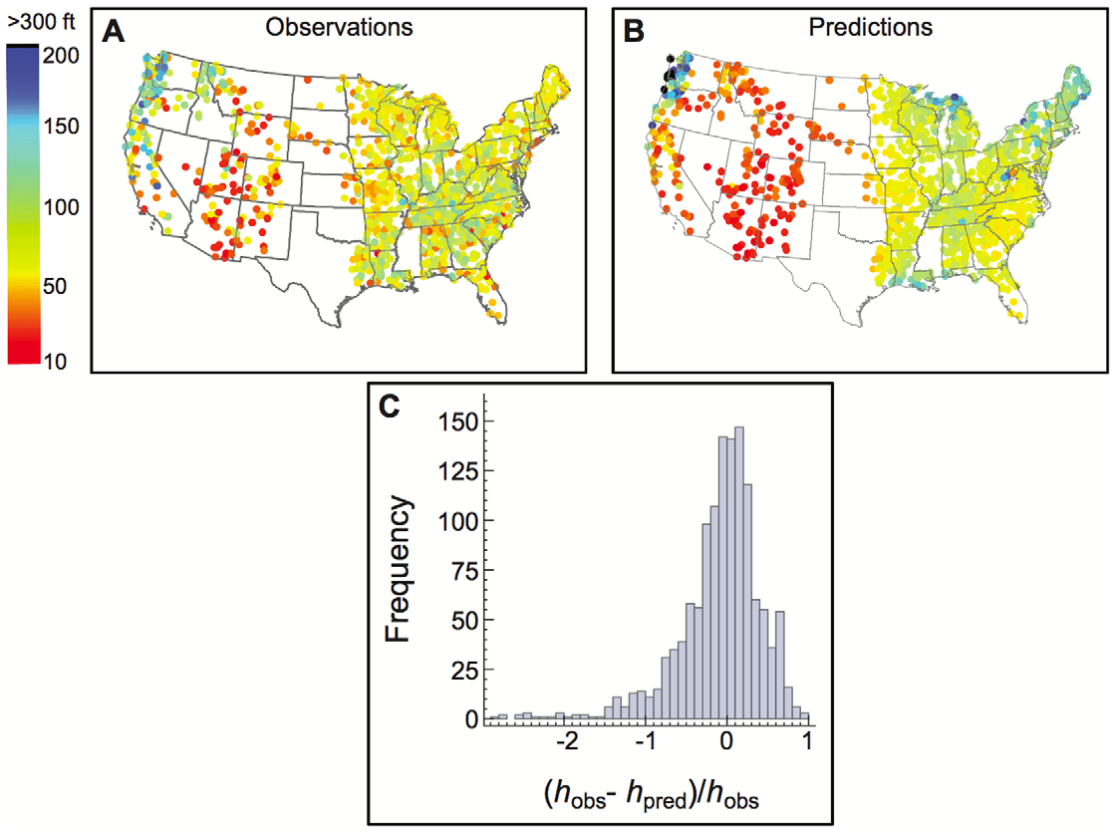
Comparisons between observed and predicted maximum tree heights. Maps of the continental United States comparing (**A**) observed and (**B**) predicted maximum heights of trees. (**C**) Histogram showing the distribution of deviations of the predicted maximum tree heights, 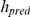, from their measured values, 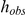, expressed in terms of the dimensionless ratio 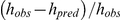. The median of the entire distribution is 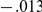 and 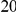 values less than 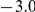 were omitted from the histogram.

Here we create a model of plant physiology that focuses almost entirely on these scaling laws which we connect with an energy budget approach and couple to environmental resources in order to calculate an important component of this variation. In particular, we incorporate the relationships between basal metabolic rate, water availability, incoming solar energy, heat loss and ambient temperature. Because the underlying scaling laws represent the average tendencies across many species we apply a single tree characterization to a variety of environments. In our framework plant diversity is encapsulated according to the average trends across many species and the scaling laws allow us to use a single parameter, tree size, to determine a range of physiological traits. We show that this model successfully predicts the local and regional variation of maximum tree heights from a small number of environmental parameters (Fig. 1). This coupling of various scaling laws also predicts more complicated relationships for tree traits such as the sigmoidal decrease in canopy albedo with increasing height. Our model can be extended to predict the variation of other plant traits and we show how stomatal density depends on local mean annual temperature.

### Previous modeling approaches

To understand the interplay between forest structure and local or regional climate it is necessary to both understand the competitive dynamics of trees within a stand and to couple tree physiology – at the individual or whole forest scale – to environmental conditions. At the regional scale, a common approach has been to focus on vegetation types coupled to atmospheric conditions. These models have successfully captured the geographic distribution of vegetation types and net primary productivity as well as environmental processes such as moisture flux and runoff [7]–[9], [31]–[35]. For understanding fine-scale forest structure explicit temporal and spatial modeling and simulation efforts are becoming increasingly accurate at capturing local forest dynamics. Several models which aim to capture local phenomena focus on the small-scale competition of trees represented either as components or patches (e.g. the JABOWA model [17], [18]) or explicitly as individually trees (e.g. SORTIE [16], [17], [19] and TASS [20]). These models predict the gap structure of the canopy [17]–[19], the species composition and diversity of a stand [16], [18], [19], the standing biomass [16]–[20], and the size distribution of trees [20] at the local scale. In the case of SORTIE, the model tracks individual trees and simulates the coupled dynamics of canopy spatial structure, crown competition, light availability, seedling recruitment, growth, and tree mortality [16], [19].

The drawback of these models is that they are computationally expensive when applied to larger regions. The more recent efforts of the perfect plasticity approximation (PPA) have used basic assumptions about the interaction of individual trees to produce macroscopic equations (analogous to those found in statistical physics) for features such as the equilibrium size distribution of trees [14]. This technique captures the average interaction between competing trees without explicitly modeling each individual and thus can be inexpensively applied to larger regions. For features which represent the average of numerous trees (e.g. total density and average height) the PPA produces very similar results to the models which explicitly track individual trees [14]. The PPA also compares well with measurements for crown characteristics such as depth and radius [11] and the temporal dynamics of stand structure, biomass and successional patterns [15].

The models discussed thus far focus primarily on either the competition dynamics within a stand or the regional coupling of environmental conditions to vegetation. The ecosystem demography model (ED) connects these two approaches in an effort to more accurately understand forest dynamics coupled to the environment at multiple scales [12]. ED relies on plant functional types as a means for capturing local forest diversity and, similar to the PPA discussed above, relates an ensemble average to the complex dynamics of individual trees including the stochastic processes of mortality and succession. ED then couples this ensemble approach with numerous environmental processes such as atmospheric conditions, fire, evapotranspiration, and carbon sequestration. ED is able to capture important local and regional phenomena such as carbon flux, standing biomass, the stock of soil carbon, or the response of productivity to changing climate [4], [5], [12], [13]. Approaches like this hold much future promise for understanding both small-scale forest structure and regional vegetation patterns as they feedback with climate. However, these models require explicit temporal simulation, and decisions about how to represent plant diversity and physiology.

### Steady-state allometric approach

Distinct from the models discussed above, the framework that we develop in this paper consists of a steady-state analytic calculation rather than a temporally and/or spatially explicit simulation. Our framework takes average local meteorology as an input and numerically calculates maximum tree height as an output. In comparison with the models discussed above our framework is not able to characterize detailed local phenomena such as temporal dynamics or species composition, but it does allow us to understand the average tendencies and constraints facing trees across different environments and this provides a useful foundation for incorporating more complicated processes.

We employ a single generalized tree across a range of environments without specific knowledge of local plant functional types commonly used in previous models [7]–[9], [34], [35]. In doing so we sacrifice accuracy at the local scale but gain a simple understanding of the average variations across environments. In the context of resources our framework lends insight into the mechanisms underlying deviations from the allometric scaling laws where, for example, we are able to show how different tree traits are suited to a given environment and predict the temperature-based variation of stomatal density (Fig. 5). These variations in turn modify the size-based scalings for an individual tree species (see Supplement S1). Ongoing work is beginning to understand departures from the basic zeroth order allometric scaling laws [24], [27], [36], [37], however, it should be noted that the zeroth order theory has yet to be coupled with environments in order to test its predictive power. Our work provides another means for expanding the basic allometric scaling laws to encompass features that are relevant to more detailed modeling efforts.

### Ecological relevance of tree height

We demonstrate the utility of our framework by predicting maximum tree height. We choose to focus on tree height because size is a natural quantity within the allometric framework and because height is an important indicator of various consequential features of a forest, such as its total resource use, biomass production rates, spatial distribution, and patterns of mortality and succession [29], [38]–[41]. For example, frequency distributions of trees follow characteristically similar relationships across forests in different regions experiencing different resource environments [29], [40], [41]. These frequency distributions follow a power-law over a large range of the data with a drop-off for the tallest trees [29], [40], [41]. This implies that the tallest trees can be used to infer the size structure of forests. Given the significance of maximum tree height our framework offers future extensions for understanding regional and global energy budgets, water and carbon cycles, temperature feedbacks, and ecosystem dynamics in response to changing environmental factors from the perspective of average physiology. It should be noted that our framework can be used to predict the variation of other plant features beyond maximum tree height such as the environmental variation of stomatal density.

Beyond its importance as a predictor of forest demographics, tree height has been shown to influence competition between individual trees for access to light [42]–[45]. However, the advantage of being taller comes with the added costs of growth and maintenance and this may set up a complicated evolutionary game between individuals [42], [43]. Maximum height has various correlations and related tradeoffs with other important plant traits [44], [45]. These include seed mass, overall growth rate, leaf mass per area, and wood density, each with environmental consequences ranging from soil resource use, to biomass production rates, to competitive dynamics within a community [44], [45]. Our framework provides insight into the environmental and physical limitations of these evolutionary dynamics.

In general, tree height is constrained by the interplay between many competing factors including resource limitations, internal metabolic constraints, overall growth rate, maturation processes, the hydrodynamic flow through vascular tubes of the branch network, its geometry and topology, and biomechanical and gravitational forces [22], [27], [28], [44]–[47], [47]–[49]. This complicated intersection of constraints is not unique to height but is a standard characteristic of most tree traits. Nevertheless, data on many properties of trees (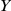) can be encapsulated and summarized in phenomenological scaling laws which typically approximate a simple power law form:
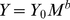
(1)where 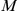 is tree mass, 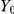 a normalization pre-factor, and 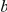 the scaling exponent. Examples include tree heights (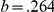), respiration rates (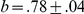 or 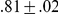), overall growth rate (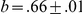), the frequency distributions of individuals (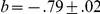), and trunk radii (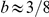) [22], [25]–[27].

These scaling laws represent the average variation of a given evolved trait across many species. Because trees have simultaneously negotiated the limitations imposed by multiple physical constraints over their complicated evolutionary trajectory, these scaling laws are likely the manifestation of multiple constraints. For example the evolved canopy structure must be both mechanically stable and able to gather sufficient solar resources in order for the tree to survive and compete. Thus considerations of either or both of these limitations may anticipate an observed empirical scaling law. By focusing on empirical scaling laws these constraints, whether known or unknown, are then implicitly incorporated into our model without needing to specify which limitations – or combination of limitations – are the most important. Both hydraulic (e.g. [46], [48]) and mechanical (e.g. [27], [47]) limitations are argued to constrain maximum tree height and our model incorporates both of these via various scaling laws including the scaling of basal flow rates and the scaling of the canopy geometry. Beyond the inherent limitations of hydraulics or mechanics, ultimately and locally, maximum tree height is governed by the availability of resources. By connecting scaling laws to an interaction with the local environment we are considering the constraints of both resources and plant structure.

## Results

### Model framework

We investigate the survival of an idealized tree with features determined primarily by its size. These features include the number of leaves, canopy shape and size, and the root mass, all of which interact with the environment via the tree's requirements for light and water (Fig. 3). Trees rely on their phloem and xylem for the internal distribution of nutrients and water. This circulation is a process of trees extracting moisture from the soil and making it available for evaporation, which drives the flow at the leaves. Accordingly, the rate of fluid flow through the vascular system has been a long-standing focus of environmental tree physiology [50], [51].

Our strategy is to compare flow rates that are constrained by resource supply with the flow rates that are required to sustain a tree of a given size in the absence of resource limitations. Both of these types of flow are governed by overall tree height according to scaling laws which relate various tree features to size. A basic assumption of our framework is that the essential tree traits required for building our predictive model scale with tree size according to approximate power laws (including isometric relationships). For many traits this is well supported by existing data. However, it should be noted that these power laws may break down for small trees where more complicated relationships hold (e.g. [27]) and some scaling exponents are known to have different values and confidence levels across different environments (e.g. [36]). These variations are beyond the scope of our efforts here. We focus on power laws because we are interested in the simplest construction of average behavior as a tool for predicting and understanding variation across species. Thus, we are testing the predictive power of the zeroth order approximation, which in this case are the widely used and studied power laws between body size and various plant traits. Future work should consider the higher order behavior of more complicated trait models.

**Figure 2:**
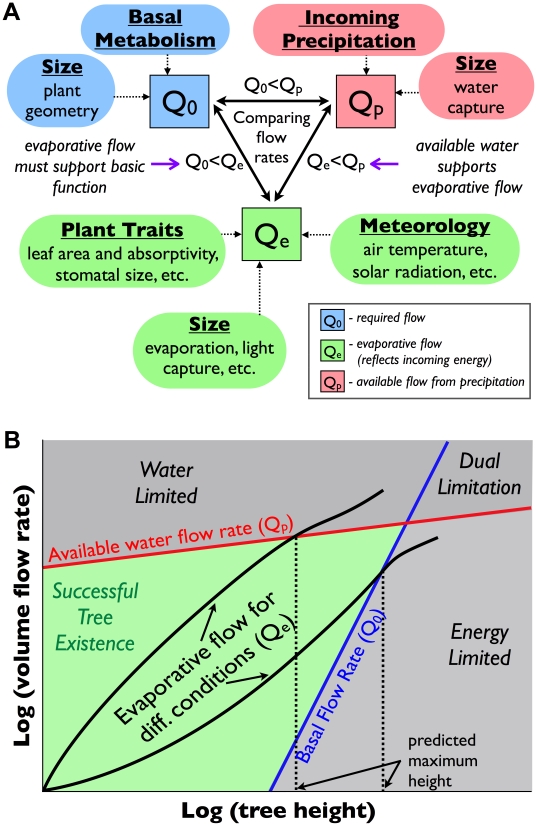
Schematics of the modeling framework. (**A**) The relationships between the required flow rate, 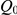, the evaporative flow rate, 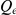, and the available flow rate, 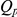, and the factors which influence them. (**B**) Limitation Diagram. Red Curve: the flow rate of available water, which is a function of precipitation and size, as described in the text. Blue Curve: the required flow rate determined from allometric scaling, which is a function of size but independent of environmental conditions. Black Curve: the calculated evaporative flow rate, which is dependent on both size and meteorological conditions. The intersection of the black curve with either of the other two determines the maximum tree height.

Scaling relationships quantify how the total *required flow rate* of water in a tree, 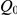, changes with overall body size in order to support its basal metabolism [28], [39], [52]. We examine two principle limitations to the flow rate in trees: available water and energy (light and heat). Energy from the environment results in an *evaporative flow rate* of water through the tree, 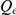, which depends on both body size as well as on meteorological conditions, including air temperature, pressure, relative humidity, and solar radiation. This evaporative flow rate, which is the actual flow rate through a tree, must be met by a sufficient *available flow rate* of water from precipitation captured by the root mass, 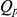, which is also dependent on body size. In addition, 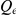 must be sufficient to support basal metabolic needs encapsulated by 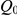. These constraints can be summarized as follows:
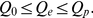
(2)Thus, 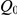 and 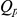 set the boundaries of acceptable flow. Maximum tree height can then be predicted by finding the largest tree for which this relationship holds. In other words, our strategy searches for trees that use energy from the environment to meet their metabolic needs without exceeding their water resources. Fig. 2A summarizes our model, highlighting the factors involved in calculating 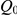, 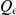, and 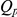.

**Figure 3:**
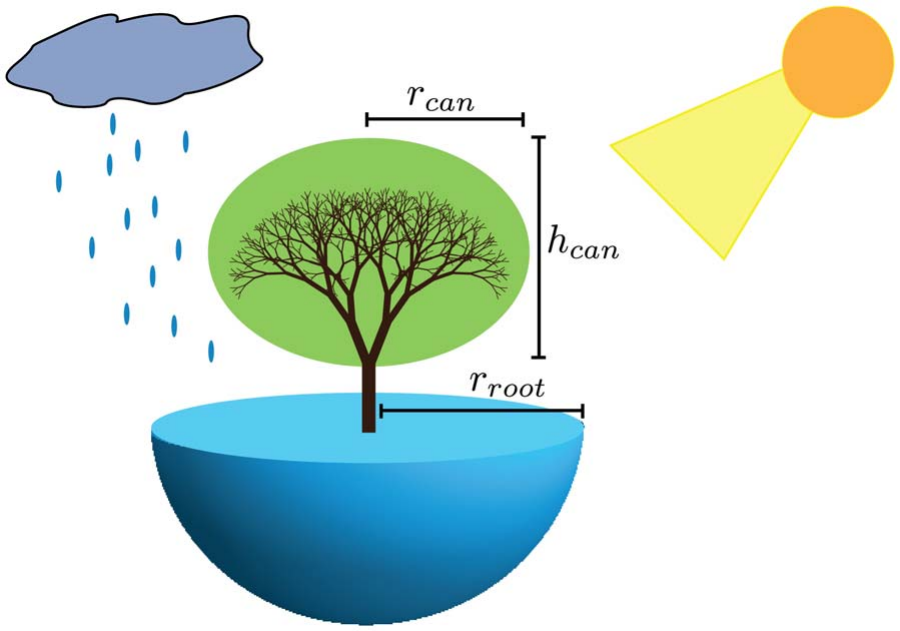
The size-based resource gathering capabilities of a tree. The above-ground canopy is shown in green and the below-ground root mass in blue. The essential dimensions of the tree are indicated, where 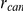 is the radius of the canopy, 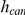 is the height of the canopy, and 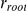 is the radius of the root mass. Each of these features scales with height, 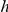, where 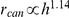 [41], 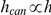 and 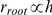. The number of leaves scales as 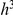 [28]. The scaling of the canopy features determines the collection of solar radiation and the heat exchange with the atmosphere, which can be used to solve for 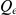. The rate of moisture absorption, 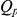, is related to the scaling of the root system and incoming precipitation. Please see Supplement S1 for a more detailed treatment of these scaling relationships along with derivations for the associated tree physiology.

Graphically, Eq. 2 implies that if we plot 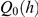 and 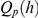 as functions of tree height, 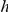, trees can only function in the region 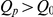 (the green-colored region of Fig. 2B). If we then plot a curve specific to a given environment, 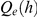, we can determine which curve, 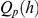 or 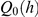, is first intersected by 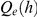 at lower 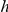. The value of 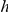 at this intersection specifies the height of the tallest possible tree. If a tree were to grow larger than this in the given environment, then its evaporative flow rate would exceed the availability of some resource. In water-rich environments lacking the appropriate incoming energy, 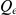 intersects 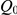 before it intersects 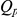, and this determines the maximum tree height. On the other hand, in water-limited environments with ample solar radiation, the reverse is true.

In order to explicitly calculate maximum tree height, we need to relate these various flow rates to tree height by invoking scaling relationships. Reference [28] provides a convenient way to relate height to several other dimensions of trees.

### Basal metabolic requirements of a plant (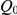)

The total basal volume flow rate of internal fluid is well approximated by
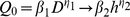
(3)where 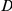 is stem diameter, 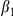 and 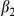 are normalization constants, and 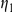 and 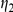 are scaling exponents [28], [29]. Empirically, best fits to data give 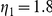, 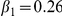 liter day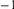 cm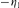 with 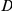 in cm, 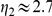 and 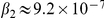 liter day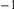 cm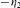 for 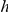 in cm [39] (see Supplement S1). In order to convert the empirical relationship in equation 3, which relates 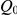 to diameter, to a relationship concerning height we employ a calculation which relates various tree dimensions such as height and diameter. For large tree sizes it has been shown in [27] that 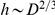 which agrees with our analysis of 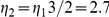 (see Supplement S1). We rely on an analytic calculation to find 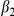 because the model in [27] includes a small tree correction to the basic power law which is outside of the scope of our stated goal. When a direct empirical relationship between two features, such as 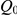 and 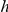, is not known we typically employ an analytic calculation in order to avoid the propagation of error resulting from the combination of two or more empirical relationships. In some situations this is not possible because there are no known analytic derivations. Our overall framework, which is simply the connection of specific set of scaling relationships, does not depend critically on these analytic calculations. For future efforts one can employ our framework and replace any given empirical or analytic scaling relationship with alternative data or calculations. All that is actually required are the phenomenological scaling relationships themselves which are, or can be, constructed from data (all parameter symbols, definitions, and values can be found in Table S1).

### Available flow rate due to precipitation (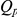)

Given an incoming rate of precipitation, and ignoring hydrology (i.e., water due to runoff, pooling, or subterranean flow and storage), the moisture available to a plant is based on the capture area and capture efficiency of the root system. The capture area for precipitation is defined by the lateral extent of the root system, which can be determined from the geometric properties of the root architecture. From the data and scaling relationships given in [22], [27], [28], [53] the radial extent of roots is approximately given by
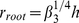
(4)with 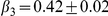 (see Supplement S1 for detailed discussion). In our model, trees have access to the total volume of precipitation that falls on the area of flat ground directly above the root system, adjusted by the absorption efficiency of the roots. This can be expressed as
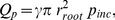
(5)where 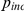 (m year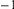) is the rate of precipitation, and 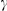 is the root absorption efficiency.

### Evaporative flow rate (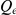)

Trees act as passive solar pumps with the rate of water escaping due to evaporation equal to the internal flow rate. Hence, 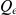 is governed by incoming energy. The basic physiological responses of tree canopies to local meteorology are well-established and are typically summarized using an energy budget [50], [51]. Although an energy budget formulation, which represents the overall conservation of energy, is conceptually simple, each individual energy flux requires a careful calculation based on the physics relevant to the appropriate tree characteristics, such as the density of stomata on a leaf and the geometry of the canopy. In Supplement S1 we provide details of these calculations which include considerations of both the tree size and environmental dependence of evaporation, radiation and conductance in the leaf and canopy microclimate. These are all governed by well-known physical laws, such as the Stefan-Boltzmann law for radiation, whose parameters have been measured or, in the few cases where they are not known, can be derived within our framework.

The basic energy budget requires that the total radiation absorption rate of a canopy, 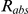, is the sum of the rates of emitted thermal radiation and the sensible and evaporative heat losses:
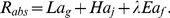
(6)Here, 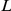, 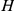, and 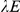 are energy fluxes (W m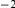): 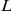 is the emitted thermal radiation, 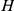 the sensible heat loss, and 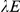 the latent heat loss with 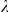 being the latent heat of vaporization for water and 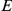 the evaporative molar flux (mol m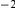 s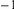) [50], [51]. The coefficients 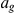, 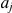, 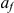 are effective areas (m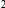) over which each heat flux occurrs and are determined by considering how the canopy architecture affects the degree to which each flux is coupled to the atmosphere.

In terms of the molar mass, 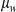 (kg mol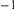), and density, 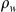 (kg m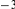), of water, the evaporative flow rate is related to 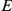 by
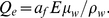
(7)From Eq. 6, we observe that the dependence of 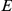, and therefore 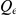, on tree height arises entirely from 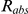 and the effective areas, 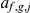, since 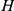, 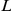, and 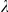 depend only on meteorological conditions. Thus, we can write
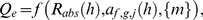
(8)where 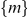 represents the set of meteorological variables.

Each effective area for heat flux has a linear dependence on the total one-sided leaf area of the canopy, 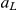, where 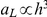. The height dependence of 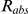 can be determined by noting that
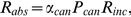
(9)where 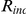 (W m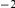) is the incoming radiation per unit area (normal to the ground), 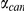 is the absorption coefficient for the canopy, and 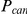 is the projected area of the canopy. Both 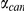 and 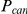 depend on tree height via the shape of the canopy and the number and distribution of leaves within that canopy. For a given incoming radiation, 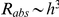 for large trees, whereas, for smaller trees, a more complex, but derivable, relation holds (please note that capital the “
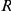
” notation refers to absorbed radiation and should not be confused with lower-case “
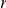
” which refers to root or canopy radii).

**Figure 4:**
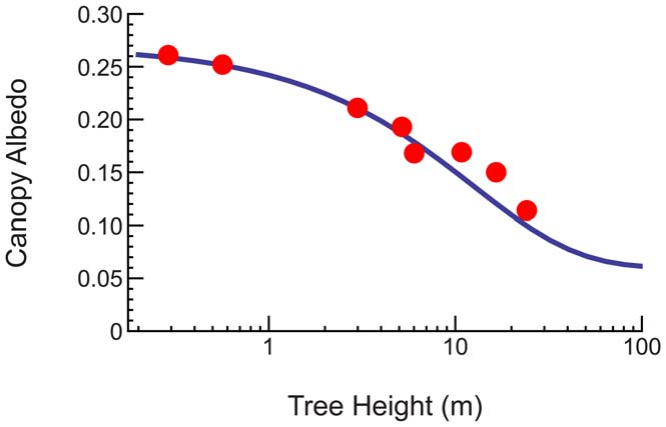
The relationship between tree height and the total albedo for the canopy of a single tree. The red points are data [67], and the blue curve is our generalized model for a tree using a soil reflection coefficient of 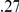 and a deep canopy reflection coefficient of 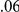 [51] (see Supplement S1). We have not included error bars here because ref. [67] does not provide them for every point.

For the average tree whose features are encapsulated in the scaling relationships, these derivations have predictive power beyond determining maximum height. For example, our model predicts the specific form of the decrease in canopy albedo with increasing tree height in excellent agreement with data, as illustrated in Fig. 4 (please see Supplement S1 for a derivation). Albedo plays a critical role in many questions related to the earth system and our model framework provides a quantitative means for linking albedo to tree heights and thereby to local resources.

Because evaporation depends on many contributing meteorological variables (solar radiation, air temperature, relative humidity, and wind-speed) and on multiple tree traits (such as average leaf size and stomatal density) it is not possible to write a simple scaling relationship for the evaporative flow rate, 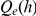. In determining 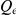 we picked representative values for tree features that entered into the calculation and used the same values across all locations. (A detailed treatment of 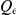 along with the parameter values used can be found in Supplement S1 in Table S1.)

### Predicting maximum tree height and other traits

To determine maximum tree heights across the continental United States, we combined meteorological data sets (see Supplement S1) to calculate the functions 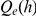 and 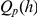 for the conditions at each location with 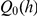 determined from Eq. 3. As discussed above, our predictions for maximum tree height are found from the first intersection of 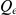 with either 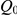 or 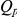. We find that 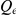 scales similarly to 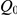 (Fig. S1) and that, in practice, the best predictions are achieved by searching for intersections of 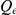 with 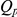 once the root absorption efficiency, 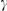, has been established (see Supplement S1).

Because tree height spans nearly two orders of magnitude, we used the relative error, 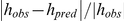, to compare our predictions, 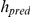, with observations, 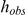, of maximum tree height. As can be seen from the figures, our model gives good agreement with observed maximum tree heights, suggesting that it does indeed capture the essential features of environmental constraints and tree physiology. Fig. 1C shows a histogram of the relative error prior to taking absolute values (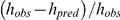) making it possible to determine over- and under-prediction. Error values are relatively narrowly distributed and the center of the distribution is close to zero. (Please see Supplement S1 and Fig. S2 for a discussion of the slight bimodal nature of this distribution.)

**Figure 5:**
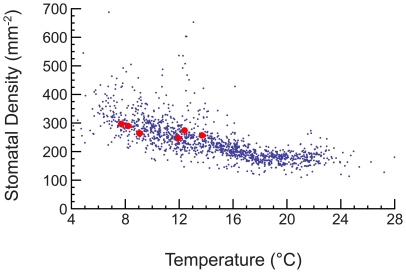
The change in the stomatal density as a function of environmental temperature. The values presented are averaged over both sides of the leaf. The blue points are predictions from our model for the optimal stomatal density in each environment, holding all other traits at the average value. The red points are observations from ref. [54]. The observations fall within the range predicted by the model.

We tend to *over*-predict maximum tree height in wet environments where there are likely competitive factors limiting tree height. Under-prediction in our model generally occurs in arid environments where trees likely have developed specialized traits which deviate from the average values we used. However, with different, more realistic trait values, such as lower stomatal density in arid environments, we find that these trees obey Eq. 2. This is to be expected as different trait values are better suited to different environments. We can expand our framework by allowing traits to vary in order to optimize maximum height while still obeying Eq. 2. For example, holding all other tree parameters constant we can find the stomatal density which maximizes the upper bound on tree height in a given environment. We observe in Fig. 5 that the optimal stomatal density that we calculate decreases with increasing average annual temperature consistent with observations [54]. We also calculated the optimal leaf size in a similar fashion and found it to decrease with increasing temperature (not shown), which is also a trend suggested by observations [55]. This type of analysis, where the model is used as a point of departure for including sub-dominant effects, including the covariation of other traits, is an important area of investigation. In Supplement S1 we conduct a similar analysis to determine the optimal allometric scaling of two plant features which we initially took to be constant, the stomatal density and root absorption efficiency. We show that incorporating these additional scaling relationships into our model can reduce the error between predictions and observations (Fig. S5). Understanding the covariation and co-optimization of various plant scalings is an important area of ongoing [24], [36], [37] and future research.

Finally, we explore the effects of environmental shifts on maximum tree heights while holding plant traits constant. Applying the simplest case of a uniform change in mean annual temperature across the United States of 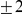 
C we can solve for the maximum height in that environment. We chose this value because 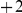 
C compares well with the conservative projections for temperature change over the next 100 years according to the frequently cited scenarios summarized by the Intergovernmental Panel on Climate Change (e.g. [56]). We find that for 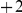 
C the average maximum height across the continental U.S. decreases by 11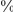 while for 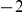 
C the average maximum height increases by 13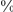 (Fig. S3).

## Discussion

It is noteworthy that our framework, which uses a general morphology and an average set of tree parameters, can consistently predict maximum tree height over a wide range of environments and tree species. At the same time, it can be easily extended to explore the specific resource tradeoffs associated with each tree trait, and thus predict environment-dependent adaptation. Various plant traits such as stomatal density and leaf size and shape have been suggested as proxies for reconstructing the paleoclimate [54], [55]. Yet some of these traits depend on multiple climatic factors. For example, stomatal density decreases with both increasing temperature and atmospheric CO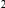 concentrations [54]. Accurate reconstruction of either temperature or CO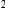 concentrations requires disentangling how each factor independently contributes to stomatal density. Our model provides simple mechanisms for interpreting how single plant traits are suited for different meteorological conditions and with this we can predict optimal plant traits for a given environment. Future work that incorporates the covariation of multiple traits may give insight into both paleo-records and the observed modern geographic variation of plant traits.

Equally important for interpreting the paleo-world is the use of allometry to reconstruct the form of paleoflora where, for example, fossilized tree trunks have been suggested as a means for reconstructing tree height [57]. Because our model makes an explicit and simple connection between local meteorology and tree size this may open up the possibility of supplementing existing proxies with trunk diameters in order to reconstruct both paleoclimate and the structure of local flora.

With respect to present day, our model can be used to anticipate potential changes in maximum tree height as a result of changes in meteorology. As maximum height is connected to local demographics and standing biomass [29], [38], [39], [41] our model may be extended to comment on how changing climate would affect these important forest features.

In short, our model has important implications for understanding tree distributions and dynamics in forests from a resource perspective and presents the possibility for understanding relationships between both paleo and modern climates and dynamic ecology. As such, it has the potential to inform important environmental issues such as migration, climate change, and carbon sequestration.

## Materials and Methods

### Scaling laws

For the empirical scaling laws used in this paper we have presented the error associated with scaling constants and exponents when the original reference provided this information.

### Height and meteorological data

For observed maximum tree heights we used the United States Forest Service's Forest Inventory and Analysis (FIA) database, which records the height and location of individual trees [58], [59]. We are interested in predicting the largest tree in an area given local meteorology. The spatial variation of meteorology can be significant over relatively short distances. Thus, it is important to pair tree sites to meteorological stations which are geographically close to one another. This ensures that the predictions are capturing the conditions experienced by the observed trees. We paired trees with meteorological stations from the National Climatic Data Center (NCDC) [60] for purposes of using station or station interpolate data. Tree-meteorology pairs were separated by no more than 100 m of elevation and 4 km of radial distance. As a result of these stringent criteria we were only able to use a small subset of trees from the FIA database.

We considered all meteorology in terms of long-term annual averages. For precipitation we used the Parameter-elevation Regression on Independent Slopes Model (PRISM) [61], [62] 30-year average (1971–2000) sampled at the location of the meteorological stations. We constructed mean temperatures for individual stations using data from the NCDC [60]. We calculated relative humidity from the PRISM 30-year average [61], [62] for mean dewpoint temperature, minimum temperature and maximum temperature using a method described in ref. [50]. For wind speeds we used data from the National Centers for Environmental Prediction (NCEP) reanalysis [63], [64]. Solar radiation data was obtained from the NREL national grid [65].

### Tree traits

Because of our focus on size and its relationship to survival in an environment we chose a single set of plant traits representative of a wide variety of tree species from different environments. This single set of traits was used across all environments to calculate 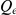. For each tree trait we examined the variation across many species, plant sizes, and environments and picked values that were representative of that variation. For several traits we checked that our values compared well to averages from the TRY database [66] which is a comprehensive collection of 65 trait databases and is representative of a large number of species and geographical regions. We picked traits that were appropriate for both angiosperms and gymnosperms, as our model does not distinguish between the two. We checked that the traits we picked gave rise to related properties, such as LAI, that were similar to observations for both angiosperms and gymnosperms. The values which we used can be found in Table S1 along with means from the TRY database.

### Sensitivity analysis

It should be noted that many empirical scaling exponents come with associated error bounds and that these exponents can vary across different environments (e.g. [36]). In addition, we have relied on several analytic derivations to inform some of the scaled tree physiology. To deal with the potential variation of exponents we have carried out a basic sensitivity analysis where we perturbed individual exponents away from the value used for our predictions and examined the shift in the median relative error between observations and the new predictions (Fig. S4) (see Supplement S1 for details).

## Supporting Information

Figure S1
**Comparisons between each of the water fluxes.** Each flux is calculated for an observed tallest tree. In each of the scatter plots the green curve is the one-to-one correspondence line. (**A**) The relationship between the available flow of water, 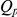, and the calculated evaporation, 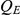. (**B**) The relationship between the theoretical basal metabolism, 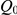, and 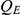. (**C**) 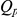 vs. 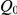.(TIF)

Figure S2
**The dependence of model error on precipitation estimates.** (**A**) Histogram of the distribution of the discrepancies between the PRISM and NARR data for rates of precipitation. (**B**) Histogram of the distribution of the discrepancies between predicted and observed tree height. Pairs of trees and station data have been removed when the error between the PRISM and NARR databases is more than 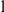 standard deviation from the mean resulting in a reduction of the slight bimodality of the error distribution.(TIFF)

Figure S3
**Predicted maximum tree height and temperature shifts.** The resulting percentage change in predicted maximum tree height given a (**A**) 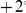
C change, (**B**) 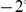
C change, (**C**) 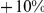 change, and (**D**) 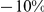 change in mean annual temperature.(TIF)

Figure S4
**Sensitivity of the model to parameter values.** The change in the median relative error between observations and predictions, 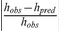, as a result of a percentage change in the given scaling exponent. The zero percentage change represents the empirical or analytic values used for the predictions in the main text.(TIFF)

Figure S5
**Optimized scaling and model error.** The change in the model predictions given an optimization in the scaling of either (**B**) stomatal density or (**C**) root absorption efficiency compared to (**A**) the original model. The red curve represents the one-to-one line. The variance of the error 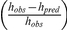 is reduced from .22 in (**A**) to .10 in (**B**) and (**C**). For all three analyses tree sites have been removed when the error between the PRISM and NARR precipitation estimates is more than 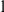 standard deviation from the mean error similar to the analysis summarized by Fig. S2. In each histogram error values less than 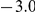 were omitted accounting for 19 values in (**A**) and 3 values in (**B**) and (**C**).(TIFF)

Supplement S1 (PDF)
