# Supplementary figures and images for: Predicting Maximum Tree Heights and Other Traits from Allometric Scaling and Resource Limitations
Source: PLoS One. 2011 Jun 13;6(6):e20551. doi: 10.1371/journal.pone.0020551 (PMC3113805; doi:10.1371/journal.pone.0020551)

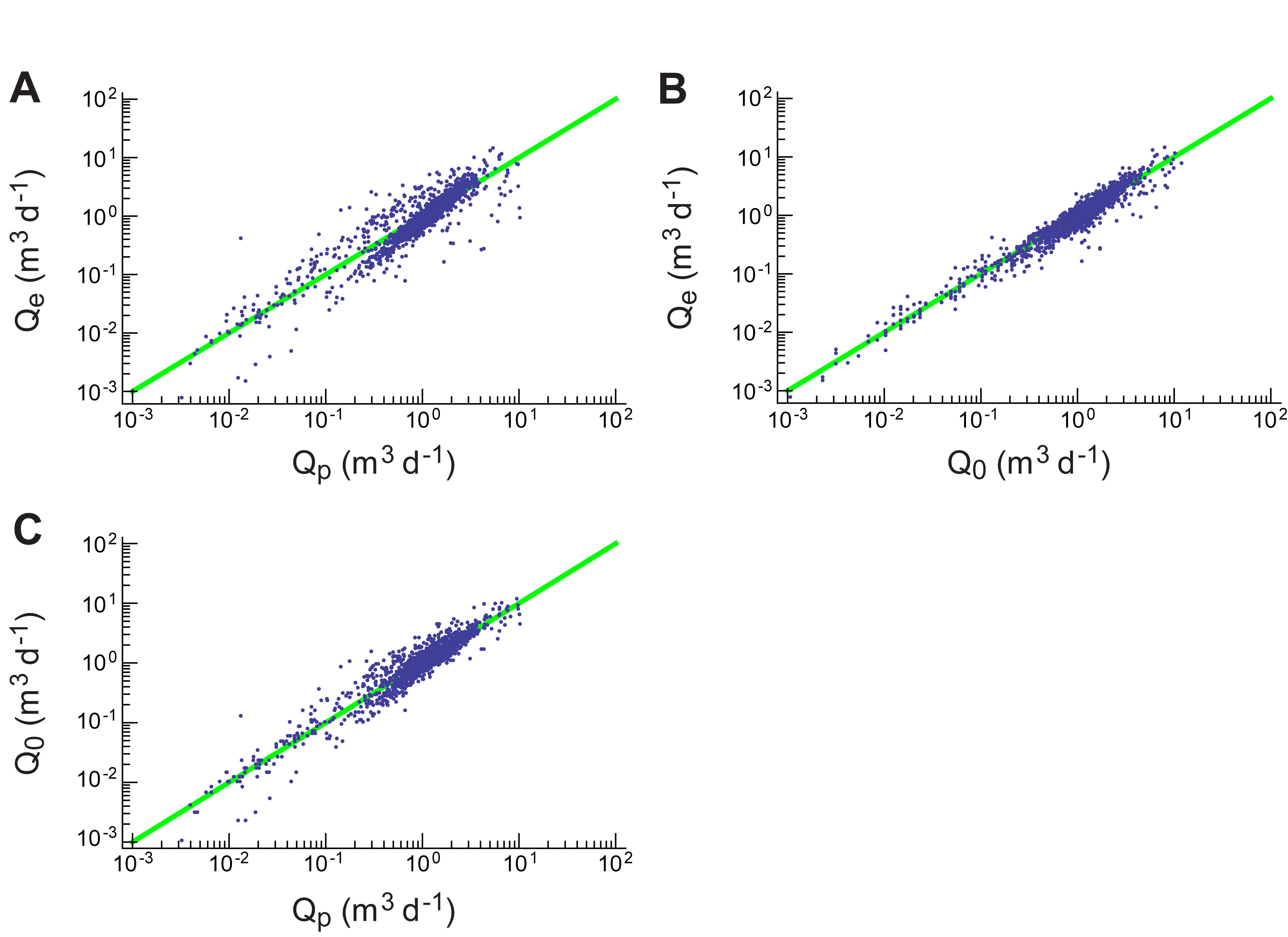

Supplement: Figure S1 — Comparisons between each of the water fluxes. Each flux is calculated for an observed tallest tree. In each of the scatter plots the green curve is the one-to-one correspondence line. (A) The relationship between the available flow of water, , and the calculated evaporation, . (B) The relationship between the theoretical basal metabolism, , and . (C) vs. . (TIF) [file pone.0020551.s001.tif]

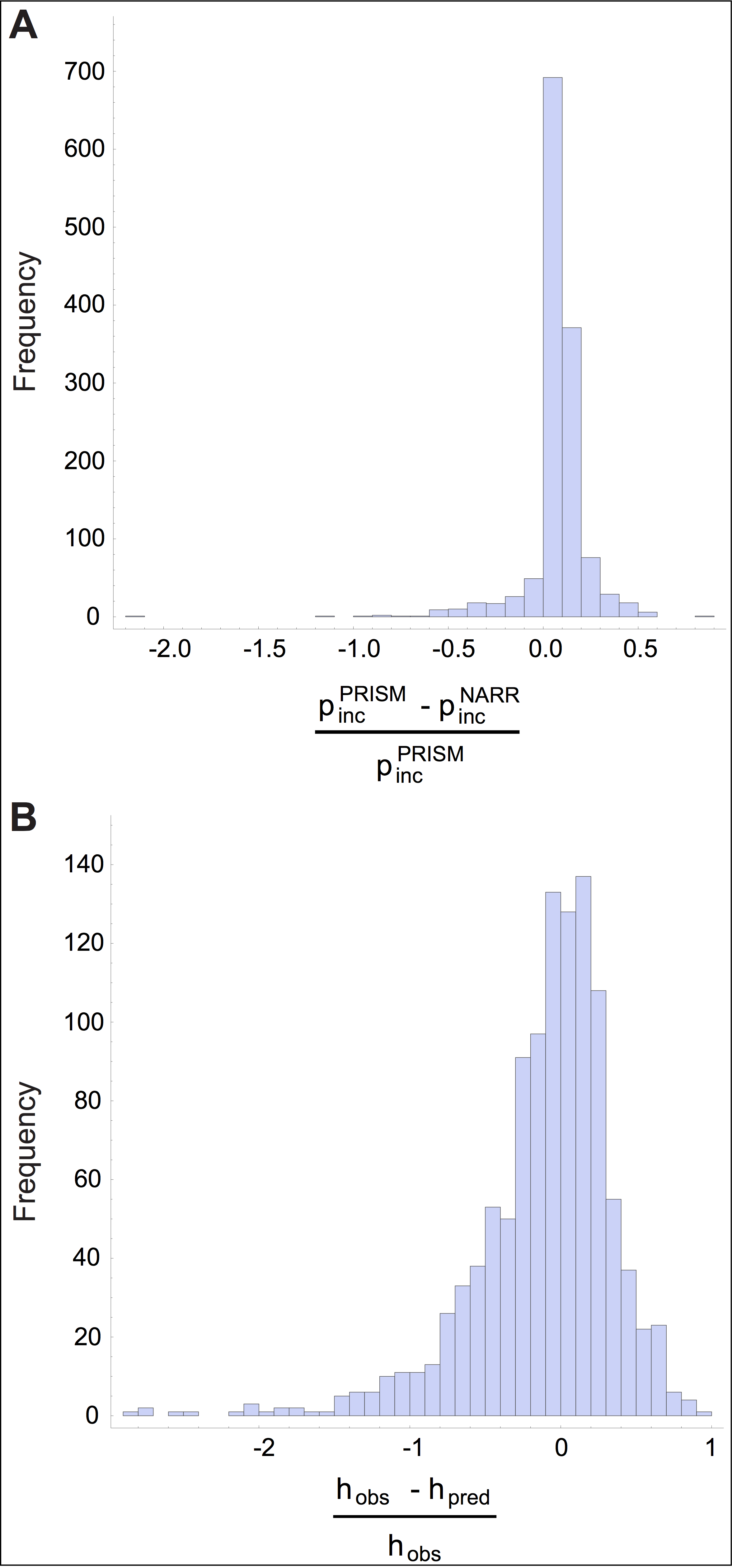

Supplement: Figure S2 — The dependence of model error on precipitation estimates. (A) Histogram of the distribution of the discrepancies between the PRISM and NARR data for rates of precipitation. (B) Histogram of the distribution of the discrepancies between predicted and observed tree height. Pairs of trees and station data have been removed when the error between the PRISM and NARR databases is more than standard deviation from the mean resulting in a reduction of the slight bimodality of the error distribution. (TIFF) [file pone.0020551.s002.tiff]

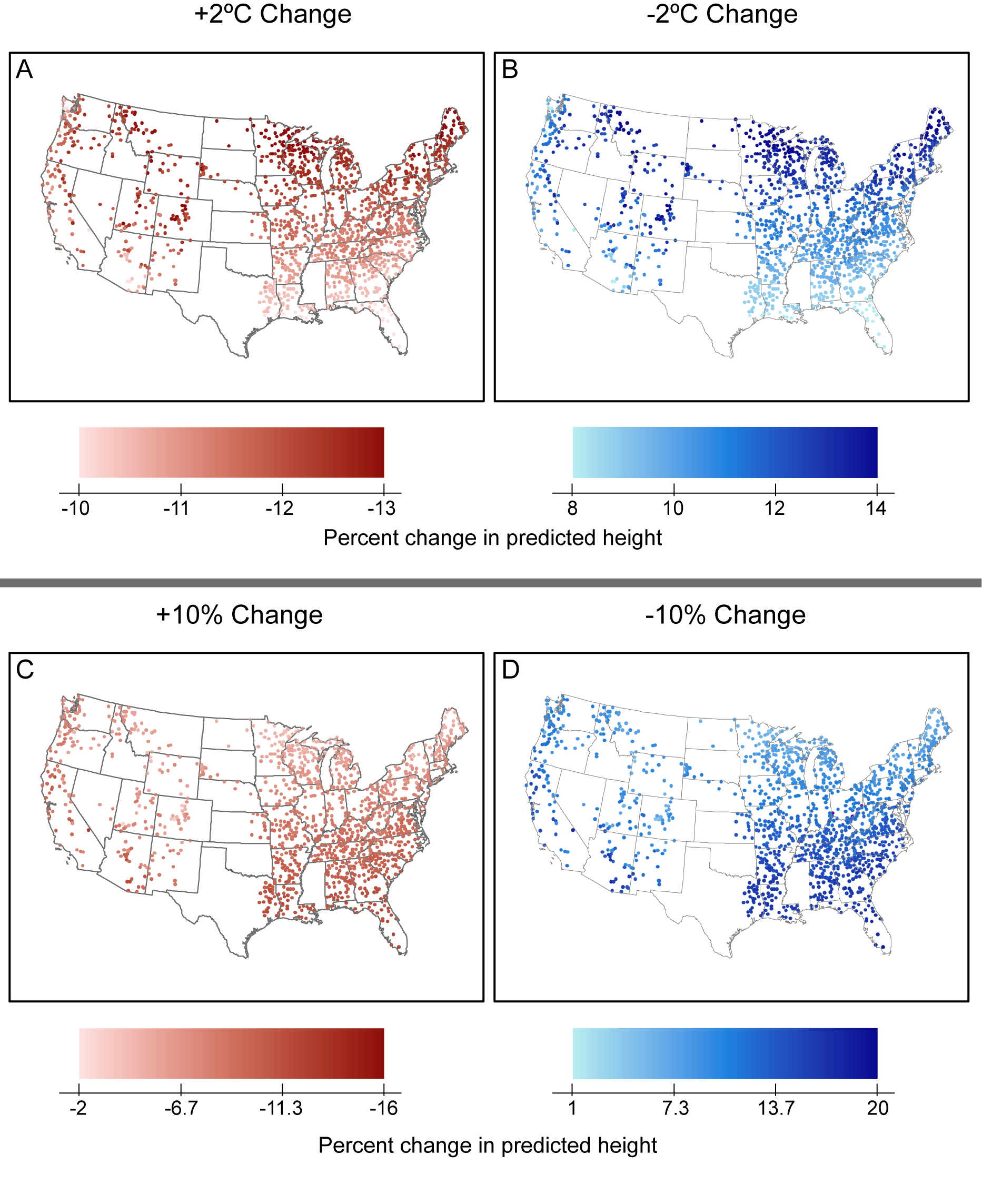

Supplement: Figure S3 — Predicted maximum tree height and temperature shifts. The resulting percentage change in predicted maximum tree height given a (A) C change, (B) C change, (C) change, and (D) change in mean annual temperature. (TIF) [file pone.0020551.s003.tif]

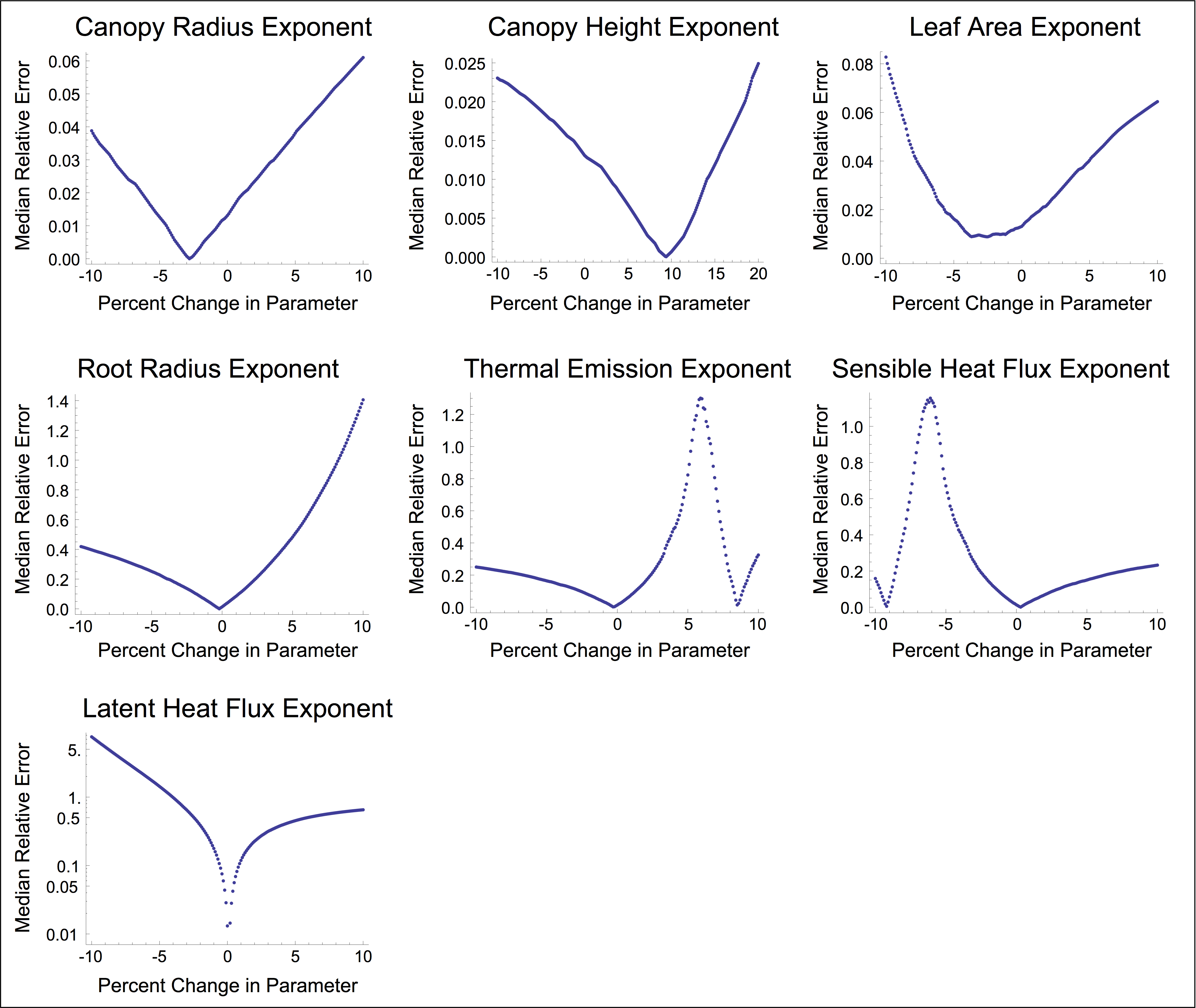

Supplement: Figure S4 — Sensitivity of the model to parameter values. The change in the median relative error between observations and predictions, , as a result of a percentage change in the given scaling exponent. The zero percentage change represents the empirical or analytic values used for the predictions in the main text. (TIFF) [file pone.0020551.s004.tiff]

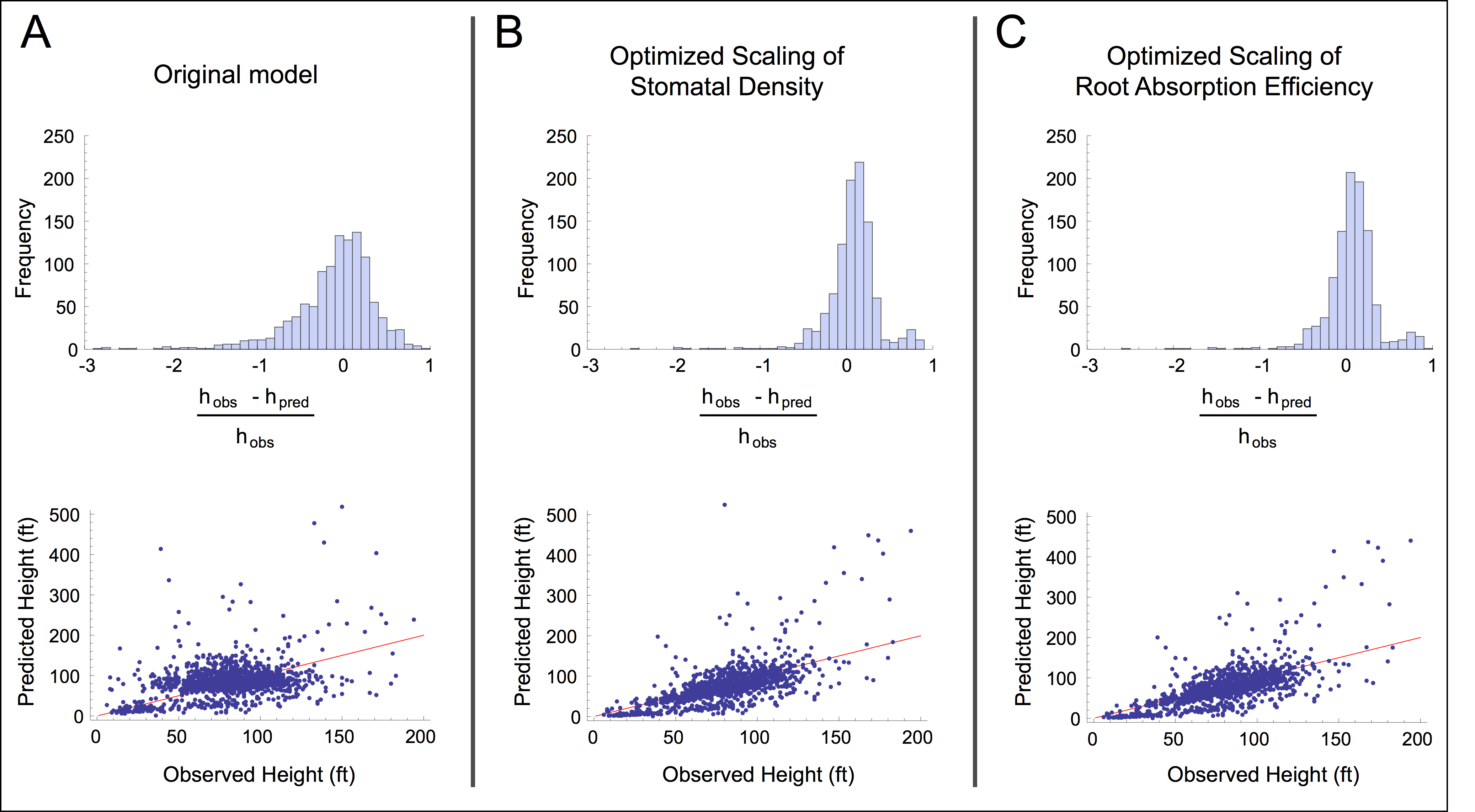

Supplement: Figure S5 — Optimized scaling and model error. The change in the model predictions given an optimization in the scaling of either (B) stomatal density or (C) root absorption efficiency compared to (A) the original model. The red curve represents the one-to-one line. The variance of the error is reduced from .22 in (A) to .10 in (B) and (C). For all three analyses tree sites have been removed when the error between the PRISM and NARR precipitation estimates is more than standard deviation from the mean error similar to the analysis summarized by Fig. S2. In each histogram error values less than were omitted accounting for 19 values in (A) and 3 values in (B) and (C). (TIFF) [file pone.0020551.s005.tiff]
