# Supplementary material for: Predicting Maximum Tree Heights and Other Traits from Allometric Scaling and Resource Limitations
Source: PLoS One. 2011 Jun 13;6(6):e20551. doi: 10.1371/journal.pone.0020551 (PMC3113805; doi:10.1371/journal.pone.0020551)
Supplement: Supplement S1 — (PDF) [file pone.0020551.s006.pdf]

Supplementary Text and Tables for "Predicting Maximum Tree Heights and Other Traits From Allometric Scaling and Resource Limitations"

Christopher P. Kempes[1,2*], Geoffrey B. West[2], Kelly Crowell[3] Michelle Girvan[2,4,5]

1 Department of Earth Atmosphere and Planetary Sciences, Massachusetts Institute of Technology, Cambridge, MA, USA

2 The Santa Fe Institute, Santa Fe, NM, USA

3 Portage Inc., Los Alamos, NM, USA

4 Department of Physics and the Institute for Physical Science and Technology, University of Maryland, College Park, MD, USA

5 Institute for Advanced Study, Princeton, NJ, USA

∗ **E-mail: ckempes@mit.edu**

Relationships within the height limitation framework

Our framework for predicting height is based on a tree's ability to collect sufficient water and sunlight to meet its basal metabolic needs without exceeding the availability of these resources. These requirements can be summarized by inequalities relating the required flow rate Q_0, the potential evaporative flow rate Q_e, and the available flow rate Q_p:

$$Q_p - Q_0 \geq 0 \tag{S1}$$

$$Q_p - Q_e \geq 0 \tag{S2}$$

$$Q_e - Q_0 \geq 0 \tag{S3}$$

Eq. S1 ensures that the tree must receive enough water to maintain its basal metabolic flow. It is through this constraint that we couple size to water resources. Eq. S2 states that precipitation must meet or exceed evaporative flow. Eq. S3 ensures that the energy a tree receives from its environment, which is translated into evaporative flow, meets its basal metabolic needs. These statements are summarized by a single condition:

$$Q_p \geq Q_e \geq Q_0. \tag{S4}$$

Thus, Q_0 and Q_p set the boundaries of acceptable flow in an environment. Maximum tree height can then be predicted by finding the largest tree for which this relationship holds. That is, our strategy searches for trees that meet their metabolic needs without exceeding their water or solar resources.

Scaling relationships

Recent work has described many allometric relationships in terms of the branching architecture of trees [1,2]. This work focuses on the hierarchy and hydrodynamic optimization of branching bundles of vascular tubes, where the mass M of a tree is related to the number of branching generations, N, by $N \propto \ln M$ [1]. This framework also describes the scaling of branch lengths and radii which, along with mass, allow for conversion between various observed allometric relationships. For a tree of height h with n daughter

branches at each branching generation, k, whose radius and length are given by r_k and l_k, respectively, it can be shown that

$$h \approx \frac{l_0}{1 - n^{-1/3}}, \quad \frac{r_k}{r_N} = n^{(N-k)(a/2)}, \quad \text{and} \quad \frac{l_k}{l_N} = \left(\frac{r_k}{r_N}\right)^{2/3a} \tag{S5}$$

where, typically, $n = 2$, and $a \approx 1$ is the branching exponent [1]. For terminal units, $r_N \approx 0.4$ mm, and $l_N \approx 4$ cm [1]. Note that plant height can also be expressed as

$$h \approx \frac{n^{N/3} l_N}{1 - n^{-1/3}} \tag{S6}$$

$$h \propto n^{N/3}. \tag{S7}$$

Many observed allometric relationships are given in terms of the basal stem diameter, D. In the relationships above $D = 2r_0$, and thus $D \propto h^{3/2}$, which agrees with the findings of reference [3] ($h \propto D^{2/3}$ for large trees). This allows us to re-write scaling relationships for stem diameter in units of tree height. For example, the basal flow rate of fluid through a tree given by

$$Q_0 = \beta_1 D^{\eta_1} \tag{S8}$$

[4] becomes

$$Q_0 = \beta_1 \left(2n^{Na/2} r_N\right)^{\eta_1} \tag{S9}$$

$$\equiv \beta_2 h^{\eta_2} \tag{S10}$$

with $\eta_2 = 3\eta_1 a/2 \approx 2.7$ and $\beta_2 = \beta_1 \left[2r_N \left(\frac{1-n^{-1/3}}{l_N}\right)^{3a/2}\right]^{\eta_1} \approx 9.2 \times 10^{-7}$ (liter day^{-1} cm$^{-\eta_2}$; for h in cm), given the parameters shown in Table 1. It should be noted that these values are found from measurements of trees *in situ* which may not be operating at the absolute basal metabolism. Future work should focus on determining the minimal requirement of a tree and this will likely adjust the value of β_1 but not the exponent η_1. This branching architecture also defines several geometric characteristics of the canopy and root systems required for calculating Q_p and Q_e.

Relationships governing the available flow Q_p

The available flow rate is determined by the incoming rate of precipitation, p_{inc}, along with the capture area and efficiency, γ, of the roots. We define the capture area in terms of the radial extent of the root system, r_{root}, so that

$$Q_p = \gamma \pi r_{root}^2 p_{inc}. \tag{S11}$$

We find that on average Q_p matches Q_0 for the tallest trees taking $\gamma = 1/3$, that is, solving for $Q_p(h_{max}) = Q_0(h_{max})$ yields a mean value of $\gamma = 1/3$, and this is the value that we use in our model. Calibrating γ in this way allows us to later solve for height using only the single limitation of Q_p. Adding variations in soil type and hydrology to calculate γ locally is an important area of future research.

The root vascular system is assumed to obey similar constraints as the canopy except that gravitational biomechanical limitations, important for canopy branches, are not important for roots. Roots may exhibit a different architecture, or overall shape, from that of the canopy, but, nevertheless, obey similar hydrodynamic and minimization constraints as the above-ground branching network. Branches and stems grow in diameter through a process of older vascular tubes becoming heartwood and new vascular tubes forming new sapwood. Since vascular tubes run the entire length of the tree, the above- and below-ground

networks are not independent. As such, branch tube formation and mortality (above-ground sapwood and heartwood formation) impose constraints on root structure. This is supported by data which show that the branching exponent a for the root system has a mean value close to unity at each branching generation, similar to that for above-ground branches [5]; (our reanalysis of data for the two species presented in Fig. 1 of ref. [5] gives $a = 1.03 \pm .33$ and $a = 1.12 \pm .45$ for $n = 2$). Combining all of these constraints results in the scaling and topology of the root system being essentially identical to that of the canopy, although the detailed architectures may be different. Thus, the total mass of the roots M_R should scale isometrically with the mass of the stems M_S, a prediction supported by the data: $M_R = \beta_3 M_S$ where $\beta_3 = 0.423 \pm 0.02$ (dimensionless) [3,6].

For optimal access to moisture the roots maximize the volume of soil occupied. The radial extent of the roots should therefore be equal to the longest tubes found in the root system. For stems, the length of a tube running from the base of the trunk to the leaves is given by $L_S \equiv h = \beta_4 M_S^{1/4}$ [1] so, given the above argument for roots, it follows that $L_R \equiv r_{root} = \beta_4 M_R^{1/4}$. Thus the radial extent of the roots is given by

$$r_{root} = \beta_3^{1/4} h. \tag{S12}$$

Later we relax these assumptions regarding the scaling of the root system with overall tree height and show how optimizing the root scaling exponent can significantly reduce the error between observed maximum tree height and our predictions.

Relationships governing the evaporative flow Q_e

Our relationship for the evaporative flow rate of a tree is determined by the interaction of the canopy with the local meteorological conditions. Here we will analyze the energy budget of the canopy, and in doing so, determine how the allometric properties of the canopy govern various heat fluxes.

Energy Budget

Our analysis of the energy budget follows ref. [7]. Conservation of energy requires:

$$R_{abs} - La_g - Ha_j - \lambda E a_f = 0 \tag{S13}$$

where R_{abs} is the total rate at which radiation is absorbed by the canopy (W) and each of the other terms is an energy flux (W m^{-2}) away from the canopy multiplied by an effective area, $a_{i=g,j,f}$, over which that flux occurs (the scaling of these areas are described below). L is the emitted thermal radiation, H is the sensible heat loss, λE is the latent heat loss with λ being the latent heat of evaporation (J mol^{-1}) and E the evaporative molar flux (mol m^{-2} s^{-1}). In the definitions below it can be seen that each of the flux terms depends on the leaf temperature, T_L (in degrees Celsius; temperatures in degrees Kelvin will be denoted using boldface $\mathbf{T}$). It is generally desirable to eliminate T_L from the system of equations in order to focus on common measurements such as the local air temperature. The Penman-Monteith method gives an approximation of T_L by linearizing each component of the energy budget in terms of $\Delta T = T_A - T_L$ and solving for T_L, where T_A is the air temperature.

Assuming $\Delta T << T_L$ and $\Delta T << T_A$ we can approximate the emitted thermal radiation as

$$L = \epsilon_l \sigma \mathbf{T}_L^4 \tag{S14}$$
$$\approx \epsilon_l \sigma \mathbf{T}_A^4 + c_p g_r \cdot (T_L - T_A) \tag{S15}$$
$$\equiv g_2 + g_1 \cdot (T_L - T_A) \tag{S16}$$

where ϵ_l is the emmisivity of the leaf, σ (W m^{-2} K^{-4}) is the Stefan-Boltzmann constant, c_p (J mol^{-1} C^{-1}) is the specific heat of air, and $g_r = 4\epsilon_l \sigma \mathbf{T}_a^3 / c_p$ (mol m^{-2} s^{-1}) is the radiative conductance (we will discuss various conductances below).

The sensible heat loss is similarly defined in terms of ΔT and is given by

$$\begin{aligned} H &= c_p g_{Ha} \cdot (T_L - T_A) && \text{(S17)} \\ &\equiv j_1 \cdot (T_L - T_A) && \text{(S18)} \end{aligned}$$

where g_{Ha} (mol m^{-2} s^{-1}) is the heat conductance of air [7].

For the latent heat loss we make the approximation

$$\begin{aligned} \lambda E &= \lambda g_v \frac{e_s(T_L) - e_s(T_A)}{p_a} && \text{(S19)} \\ &\approx \lambda g_v \frac{1}{p_a} \frac{de_s(T_A)}{dT_A} \cdot (T_L - T_A) + \lambda g_v \frac{D_v}{p_a} && \text{(S20)} \\ &\equiv f_1 \cdot (T_L - T_A) + f_2 && \text{(S21)} \end{aligned}$$

where g_v (mol m^{-2} s^{-1}) is the vapor conductance, T_D is the dew point temperature, p_a is the air pressure (kPa), and $D_v \equiv e_s(T_A) - e_s(T_D)$ (kPa) is the vapor pressure deficit, and

$$e_s(T_A) = b_1 \exp\left(\frac{b_2 T_A}{b_3 + T_A}\right) \quad \text{(S22)}$$

is the Tetans formula for saturation vapor pressure (kPa) with $b_1 = .611$ (kPa), $b_2 = 17.502$ (dimensionless), and $b_3 = 240.97$ (°C) [7].

Solving this system of equations we obtain

$$T_L - T_A = \frac{R_{abs} - f_2 a_f - g_2 a_g}{f_1 a_f + g_1 a_g + j_1 a_j}, \quad \text{(S23)}$$

which, using the approximation in Eq. S21, leads to an equation for the evaporative flux

$$E = \frac{f_1}{\lambda}\left[\frac{R_{abs} - f_2 a_f - g_2 a_g}{f_1 a_f + g_1 a_g + j_1 a_j}\right] + \frac{f_2}{\lambda}. \quad \text{(S24)}$$

The total volume flow rate, Q_e, is obtained by multiplying E_{can} by the area a_f, and then converting from a molar rate to a volume flow rate. Thus $Q_e = a_f E \mu_w / \rho_w$ given the molar mass, μ_w (kg mol^{-1}), and density, ρ_w (kg m^{-3}), of water. This can be written in full as

$$Q_e = a_f \frac{\mu_w}{\rho_w}\left(\frac{f_1}{\lambda}\left[\frac{R_{abs} - f_2 a_f + g_2 a_g}{f_1 a_f + g_1 a_g + j_1 a_j}\right] + \frac{f_2}{\lambda}\right). \quad \text{(S25)}$$

Allometric canopy geometry and solar radiation

In this section we derive the rate of absorbed solar radiation, R_{abs}, a necessary component of Q_e. This absorption rate represents the relationship between available solar resources, plant structure, and, ultimately, tree height.

The collection of solar radiation by a plant can be described by coefficients for canopy transmission, τ, canopy reflection, ξ_c, and soil reflection ξ_s all of which can be encapsulated as the canopy absorption coefficient

$$\alpha_{can} = 1 - \xi_c - (1 - \xi_s)\,\tau \quad \text{(S26)}$$

[8]. A commonly used model [7,8], which we find connects easily to the branching architecture discussed above, assumes that leaves are uniformly distributed over a spheroidal canopy of surface area, S_{can},

defined by the semi-axes $h'_{can} = h_{can}/2$ and r_{can}. In that case, the transmission coefficient is given by a classic absorption formula

$$\tau = e^{-\sqrt{\alpha}K(\psi)LAI} \tag{S27}$$

[7, 8], where α is leaf absorptivity and LAI is the leaf area index, a measure of the total leaf area per unit ground below the canopy, which is given by

$$LAI = \frac{a_l n^N}{\pi r_{can}^2}. \tag{S28}$$

The extinction coefficient, $K(\psi) = 2P_{can}/S_{can}$, describes the ratio of canopy projected area, P_{can}, to canopy surface area, S_{can}, and is a function of the solar zenith angle ψ. Given a spheroidal canopy, it follows that

$$P_{can} = \pi r_{can}(r_{can}\cos\theta + h'_{can}\sin\theta\tan -\psi), \tag{S29}$$

and

$$S_{can} = 2\pi\left(r_{can}^2 + \frac{r_{can}\left(h'_{can}\right)^2 \arcsin\left[\frac{\sqrt{(h'_{can})^2 - r_{can}^2}}{h'_{can}}\right]}{\sqrt{\left(h'_{can}\right)^2 - r_{can}^2}}\right), \tag{S30}$$

[8, 9] where $\theta = \arctan\left(-h'_{can}/r_{can}\tan\psi\right)$ is the angle defining the point of ray tangency on the canopy surface and $h'_{can} = h_{can}/2$.

The transmission coefficient, τ, can be calculated by combining the above equations. The total solar radiation captured by the canopy is then given by

$$R_{abs} = \left[1 - \xi_c - (1 - \xi_s)\,\tau\right] P_{can} R_{inc}, \tag{S31}$$

where R_{inc} (W m^{-2}) is the incoming radiation per unit area (normal to the ground), and ξ_c and ξ_s are described below.

Heat flux areas

Our canopy energy budget is derived by scaling up from the budget for a single leaf whose properties are assumed invariant across trees of different size. In this picture, changes in energy fluxes are predominantly due to allometric relationships for the canopy shape and number of leaves. Leaves are assumed to act in parallel, and thus the total area for sensible heat loss is $a_j = 2a_L$, where a_L is the total one-sided area of all the leaves on the tree, given by $a_L = a_l n^N \propto h^3 \propto M^{3/4}$ [1]. The evaporative flux, on the other hand, occurs over the total area of the stomatal openings in the leaves, $a_f = 2a_L\delta_s a_s$, where δ_s is the density of stomata on a leaf, and a_s is the area of a single stoma.

If L is the thermal radiative flux emanating from the tree, then a good approximation for the effective radiative surface area is the total one-sided leaf area, so $a_g \approx a_L$. Since $S_{can} \sim h^2$, whereas $a_L \sim h^3$, leaves will overlap on the canopy surface for sufficiently large trees so that the above approximation for a_g becomes less accurate. We tested other schemes for a_g, such as using S_{can}, or the minimum of S_{can} and a_L, and found similar results to the ones presented here.

Scaling of the canopy radius and height

In previous work, it has been shown that the canopy radius scales as $r_{can}^3 \propto r_0^2$ [10], or

$$r_{can} \propto n^{N/3} r_N^{2/3} \propto h \tag{S32}$$

which follow from Eqs. S5 and S6. The scaling with height is consistent with empirical data where it has been shown that $r_{can} = \beta_5 h^{\eta_3}$, with $\eta_3 = 1.14$, and $\beta_5 = 35.24$ (cm m$^{-\eta_3}$; for h in meters) (see SI of ref. [11]).

Unlike r_{can}, we were unable to locate data characterizing the scaling of h_{can} with total tree height, h. Following ref. [12], we assume that the canopy scales isometrically so that $h_{can} = \beta_6 h$, where β_6 is a constant. A lower bound for the canopy height can be obtained from a configuration in which all of the branches of the tree emanate directly upwards from the top of the trunk leading to $h_{can} \geq h - l_0$; clearly, an upper bound is given by the total height of the tree: $h_{can} \leq h$. Since, $h \approx l_0/\left(1 - n^{-1/3}\right)$, we obtain the bound

$$h \geq h_{can} > n^{-1/3} h \approx 0.79\ h \tag{S33}$$

or

$$1 \geq \beta_6 > n^{-1/3} \approx 0.79 \tag{S34}$$

for $n = 2$. Note, further, that, from data the diameter of the canopy, $d_{can} = 0.7h^{1.1}$, indicating that, generally speaking, canopy height exceeds canopy diameter, $h_{can} > d_{can}$, suggesting that the canopy forms an approximate prolate spheroid. For the discussion presented in the text, we took $\beta_6 = 1$. Varying β_6 between 0.8 and 1 slightly shifted the mean of the distribution in Fig. 1C of the main text.

Canopy Radiation Coefficients

Above, we discussed the derivation of τ, which is one of the radiation coefficients involved in calculating the total radiation absorbed by a canopy. The other two coefficients correspond to the canopy and soil reflection, respectively, ξ_c and ξ_s. The soil reflectivity can be taken as a constant value which is given in Table 1, but the canopy reflectivity is expected to change based on the size and scattering properties of the canopy. It has been shown that the canopy reflection, as a function of the leaf area index, LAI and extinction coefficient $K(\psi)$ is well approximated by

$$\xi_c = \frac{\xi_c^* + f e^{-2K(\psi)LAI}}{1 + \xi_c^* f e^{-2K(\psi)LAI}}, \tag{S35}$$

where

$$f = \frac{\xi_c^* - \xi_s}{\xi_c^* \xi_s - 1} \tag{S36}$$

and ξ_c^* is the deep canopy reflection coefficient, which is constant [7, 8]. For small LAI, Eq. S35 is approximately the ground reflectivity ξ_s, and for large LAI, it is ξ_c^*.

Combining equations S26, S27, and S35 it is possible to determine the total absorption coefficient of the canopy and this is the calculation used to produce the curve in Fig. 4 of the main text.

Canopy conductances

Each of the above terms depends on the conductances of heat, vapor, and radiation which are described below. Assuming that the canopy surface is exposed to forced laminar convection, the heat conductance in air is given by

$$g_{Ha} = \frac{0.664 \hat{\rho}_a D_H Re_a^{1/2} Pr_a^{1/3}}{d} \quad (\text{mol m}^{-2}\ \text{s}^{-1}) \tag{S37}$$

where $Re_a = ud/\nu_a$ is the dimensionless Reynolds number for air, $Pr_a = \nu_a/D_H$ is the dimensionless Prandtl number for air, D_H (m^2 s^{-1}) is the heat diffusivity, $\nu_a(T_a)$ (m^2 s^{-1}) is the kinematic viscosity of

air, u (m s^{-1}) is the wind speed, $d = 0.81\left(2\left(a_l/\pi\right)^{1/2}\right)$ (m) is the characteristic dimension of a circular leaf of area a_l (m), and $\hat{\rho}_a$ is the molar density of air given by the Boyle-Charles law as

$$\hat{\rho}_a = 44.6\frac{p_a}{101.3}\frac{273.15}{\mathbf{T_A}} \quad (\text{mol m}^{-3}) \tag{S38}$$

for Kelvin temperature $\mathbf{T_A}$ and air pressure $p_a = 101.3e^{-A/8200}$ (kPa) at altitude A (m) [7].

We assume that the canopy is in series with the atmospheric boundary layer, so $g_v = 1/\left(\frac{1}{g_{vL}} + \frac{1}{g_{va}}\right)$, where g_{vL} and g_{va} are, respectively, the vapor conductances for the leaf and atmosphere. Assuming forced laminar convection, as before, the boundary layer conductance is

$$g_{va} = \frac{0.664\hat{\rho}_a D_v Re_a^{1/2} Sc_a^{1/3}}{d} \quad (\text{mol m}^{-2}\ \text{s}^{-1}), \tag{S39}$$

where $Sc_a = \frac{\nu_a}{D_v}$ is the dimensionless Schmidt number, and D_v (m^2 s^{-1}) is the diffusivity for water vapor in air. The leaf conductance is calculated by considering the stomata as acting in parallel. We model a single stoma as a cylinder of depth z_s (m) in which case Fick's law gives

$$g_{vs} = \frac{\hat{\rho}_a D_v}{a_s(0)\int_{z_0}^{z_b}\frac{dz}{a_s(z)}} = \frac{\hat{\rho}_a D_v}{z_s} \quad (\text{mol m}^{-2}\ \text{s}^{-1}) \tag{S40}$$

for the conductance of a single stoma. Summing over the entire leaf area, the leaf conductance is given by

$$g_{vL} = g_{vs} a_s \delta_s \quad (\text{mol m}^{-2}\ \text{s}^{-1}), \tag{S41}$$

where a_s (m^2) is the area of the opening of a single stoma, and δ_s (stomata m^{-2}) is the stomatal density on a leaf.

Trait values

For each tree trait we examined the literature in order to gain a sense of the variation of each trait across many species, plant sizes, and environments and we picked values that were representative of that variation. For several of these values we were able to check for agreement with means from the TRY database [13] which is a collection of multiple databases containing global values for various plant traits across numerous environments and species. For each trait there are several ways to analyze the distribution of trait values produced by the TRY database. The first is to take the straight mean, which we present in Table 1. The second is to recognize that most of these traits appear to be lognormally distributed in which case it is possible to logarithmically transform the data, find the transformed mean, and then take the exponential of that value. Formally this is the median assuming a lognormal distribution and these values are also given in Table 1. These two analyses typically provide a range of possible representative values for each trait, and the values that we used fall within this range.

Relationships between Q_0, Q_e, and Q_p for observed tallest trees

We tested the model by looking at observed maximum tree heights in different environments to check whether they do indeed adhere to the given resource constraints governing the required, evaporative, and available flow rates $Q_p(h_{obs}) \geq Q_e(h_{obs}) \geq Q_0(h_{obs})$. We calculate each of these quantities using observed maximum heights and measured environmental conditions. For trees which have grown to reach the upper bound on height in a given environment we would expect that $Q_p(h_{max}) = Q_e(h_{max}) = Q_0(h_{max})$. Figure S1A-C shows the resulting pair-wise relationship between each of these quantities.

The derived data generally fall along the green line which represents equality between the two quantities being considered. In each plot the scaling of the two flows is independent of the choice of γ, but the overall intercept will depend on this value.

Detailed examination of predicted vs. observed tree heights

The error between predictions and observations, as shown in Fig. 1C of the main text, is slightly bimodal with the secondary mode corresponding to situations where we *under*-predict tree height. Most trees in the under-prediction mode are found in mountainous regions (elevations greater than 1000m) where there is often high spatial variability in precipitation. Thus the interpolated meteorology may not be representative of the environment experienced at the tree location. We tested this hypothesis by comparing precipitation estimates from the Parameter-elevation Regression on Independent Slopes Model (PRISM) [14, 15] to estimates from the North American Regional Reanalysis (NARR) [16, 17]. Large discrepancies between the two databases may indicate either differences in the modeling approach used to assimilate individual station data or high spatial variability in the meteorology. Fig. S2A shows the distribution of the discrepancies between the two analyses. The two data-sets are typically in good agreement, with the PRISM data generally being a little larger than the NARR data. When we removed locations where the error between the two precipitation estimates was more than one standard deviation away from the mean then the under-prediction mode was diminished (Fig. S2B) in the distribution of error between observations and predictions of maximum tree height.

Temperature Shifts

A central question in ecology is how ecosystems respond to environmental shifts. Temperature plays a dominant role in controlling evaporative rates in our framework and here we investigate how our predictions for maximum tree height respond to changes in mean annual temperature. We consider a fairly large change in mean annual temperature of ± 2 °C. The maps in Figures S3A and B illustrate the resulting percentage change in predicted maximum height. Averaging over the entire continent the maximum tree height decreases by 11% for a $+2$ °C shift while maximum height increases by 13% for a -2 °C shift. For an increase in temperature nearly all predicted maximum heights will decrease, although the percentage change varies regionally, and for a decrease in temperature nearly all predicted heights will increase.

Looking across different environments we find that warmer environments are less sensitive to a ± 2 °C shift than colder environments, hence the strong gradient in percentage change with latitude and/or elevation. The northern and mountainous regions are more sensitive to an absolute shift in temperature. However, it should be noted that a ± 2 °C shift in a colder environment represents a much larger percentage change in mean annual temperature compared with a warmer environment. Another analysis which accounts for this effect is to change temperature by $\pm 10\%$ in every environment. Doing this we find that warmer environments are more sensitive to a constant percentage change than colder environments (Figs. S3C and D). For the $+10\%$ change in mean annual temperature we find that the average change in maximum tree height is -8% across the entire continental United States. For the -10% change maximum heights change by an average of $+9\%$.

Parameter Sensitivity

It should be noted that the empirical exponents used in this study are fits to data and are accompanied by some amount of error. In addition there are several assumptions or derivations that we have made regarding the scaling of plant features for which there is no data. An important question is how robust

our model is to small deviations in the values of these empirical or analytic exponents. Yet understanding the covariation of all of the exponents such that they all are in agreement with data is an interesting and complicated problem (see refs. [18–20]). For the suite of exponents involved in this paper the mechanisms of covariation are simply not known, and we do not have data for each property. Thus a full-scale, empirically-realistic sensitivity analysis to parameter combinations is not possible. Given these limitations we can however examine the effects of perturbing each exponent independently of the others. Figure S4 shows changes in the median relative error, $\left|\frac{h_{obs}-h_{pred}}{h_{obs}}\right|$, of our predictions given a percent change in the dominant exponents of our model (including analytic assumptions or results). The dominant exponents are those which control water acquisition (the root radius scaling, r_{root}), and those which regulate the canopy energy budget via its geometry and heat flux areas (canopy height, h_{can}, and radius r_{can}, and the thermal, sensible, and latent heat flux areas, a_g, a_j, a_f respectively). Our original assumption is that all heat flux areas scale according to the scaling of leaf area (see above). In Figure S4 we test changes to this leaf area scaling as well as changes to the scaling of each heat flux area individually. In each plot zero represents the parameter value used in our model, and it can be seen that for each parameter there is a minimum in the median relative error (Fig. S4). This minimum often does not occur at the value used for our original predictions, revealing how our model could be optimized to more accurately predict data. In the future it may be possible to optimize all exponents simultaneously in order to achieve greater predictive power. However, our goal here is to test the zeroth order theory based on the available observations.

For the canopy height and radius and the scaling of the total leaf area, small changes to the exponents result in small changes to the median relative error. However, the median relative error is very sensitive to the scaling of the root radius which directly affects moisture gathering, and to differences in the scaling of the heat flux areas compared to the overall leaf area. The thermal and sensible heat flux areas contain multiple minima in the relative error and these two parameters have competing effects with one another illustrating the complicated connection between exponents and the importance of parameter covariation for a true assessment of sensitivity. Each heat flux exponent represents a physiological response to the environment, and given the complicated connection of these exponents it is of future interest to test whether our framework can be used to predict changes in exponents across environments as observed by [18].

Improving predictions using parameter optimization

From Figure 1C of the main text, it is apparent that, despite overall good agreement with the model predictions, there are significant outliers representing deviations between observations and our predictions. This is hardly surprising, given the many potential sources of error such as a possible mismatch between meteorological measurements and the actual conditions experienced by the observed trees (see discussion regarding Fig. S2), and processes such as logging, mortality, and fire which are not included in the scope of our analysis. Additionally, the measured tallest trees may not have reached the environmentally-determined upper bound. As we have seen in Figure 5 of the main text, our model anticipates the observed environmental dependence of optimal plant traits and this implies that using only a single set of plant traits across all environments may also be a source of error. Finally, some of our assumptions regarding unmeasured scaling relationships may be inaccurate and contribute to error within the predictions. Our goal was to capture the central tendencies, or average behavior, given a set of known (both observed and theoretically motivated) scaling relationships and average plant traits. However, here we show how our predictions can be improved by finding additional scaling relationships which optimize the model.

In the main text we solved for the stomatal density which maximized height at a given location and set of environmental conditions (Fig. 5). Looking across all of the tree sites we found a relationship between stomatal density and temperature. Similarly, we can solve for the stomatal density, δ_s, which gives

$Q_P(h_{obs}) = Q_E(h_{obs}, \delta_s, \{m\})$ for every observed tallest tree (given the local meteorological conditions $\{m\}$). Doing this we find that stomatal density should scale with tree height as $\delta_s \propto h^{-.71}$ (assuming a constant stomatal area). Incorporating this scaling into our model then improves our predictions as illustrated in Figure S5B. In our model we also use a constant value of the root absorption efficiency γ and we can also optimize the scaling of this trait alone where we find that $\gamma \propto h^{-.78}$ also reduces the error in the predictions (Fig. S5C). This demonstrates the potential utility of the model in anticipating scaling laws for which measurements do not already exist. However, it should be noted that these two analyses optimize scaling with respect to only a single tree feature one at a time, and, for comparison with future data, it is likely necessary to co-optimize all exponents of relevance. This analysis is an example to illustrate that adding additional scalings to the model can improve predictability and reduce the variance of the deviations; however finding the realistic set of exponents subject to multiple limitations is a subject of ongoing research and requires a more advanced optimization study

References

1. West GB, Brown JH, Enquist BJ (1999) A general model for the structure and allometry of plant vascular systems. Nature 400: 664–667.

2. West GB, Brown JH, Enquist BJ (1997) A general model for the origin of allometric scaling laws in biology. Science 276: 122–126.

3. Niklas KJ, Spatz HC (2004) Growth and hydraulic (not mechanical) constraints govern the scaling of tree height and mass. Proceedings of the National Academy of Sciences of the United States of America 101: 15661–15663.

4. Enquist BJ, Brown JH, West GB (1998) Allometric scaling of plant energetics and population density. Nature 395: 163–165.

5. Salas E, Ozier-Lafontaine H, Nygren P (2004) A fractal root model applied for estimating the root biomass and architecture in two tropical legume tree species. Annals of Forest Science 61: 337–345.

6. Niklas KJ (2004) Plant allometry: is there a grand unifying theory? Biological reviews 79: 871–889.

7. Campbell GS, Norman JM (1998) Introduction to environmental biophysics, second edition. Springer Verlag.

8. Monteith JL, Unsworth M (2008) Principles of environmental physics, second edition. Academic Press.

9. Hilbert D, Cohn-Vossen S (1999) Geometry and the Imagination. American Mathematical Society.

10. Niklas KJ, Spatz HC (2006) Allometric theory and the mechanical stability of large trees: proof and conjecture. American journal of botany 93: 824–828.

11. Enquist BJ, West GB, Brown JH (2009) Extensions and evaluations of a general quantitative theory of forest structure and dynamics. Proceedings of the National Academy of Sciences 106: 7046–7051.

12. Enquist BJ, West GB, Brown JH (2009) A general quantitative theory of forest structure and dynamics . Proceedings of the National Academy of Sciences 106: 7040–7045.

13. TRY database, www.try-db.org, accessed 26 July 2010.

14. Daly C, Neilson RP, Phillips DL (1994) A statistical-topographic model for mapping climatological precipitation over mountainous terrain. Journal of applied meteorology 33: 140–158.

15. PRISM Climate Group, Oregon State University, http://www.prismclimate.org, accessed 16 December 2007.

16. Mesinger F, DiMego G, Kalnay E, Mitchell K, Shafran PC, et al. (2006) North American regional reanalysis. Bulletin of the American Meteorological Society 87: 343–360.

17. NCEP/NCAR North American American Regional Reanalysis Archive, http://dss.ucar.edu/pub/narr/, accessed 20 March 2009.

18. Price C, Ogle K, White E, Weitz J (2009) Evaluating scaling models in biology using hierarchical Bayesian approaches. Ecology letters 12: 641–651.

19. Price C, Gilooly J, Allen A, Weitz J, Niklas K (2010) The metabolic theory of ecology: prospects and challenges for plant biology. New Phytologist 188: 696–710.

20. Price C, Enquist B, Savage V (2007) A general model for allometric covariation in botanical form and function. Proceedings of the National Academy of Sciences 104: 13204–13209.

21. Royer DL, Wilf P, Janesko DA, Kowalski EA, Dilcher DL (2005) Correlations of climate and plant ecology to leaf size and shape: potential proxies for the fossil record. American Journal of Botany 92: 1141–1151.

22. Falster DS, Westoby M (2003) Leaf size and angle vary widely across species: what consequences for light interception? New Phytologist 158: 509–525.

23. Ackerly DD, Reich PB (1999) Convergence and correlations among leaf size and function in seed plants: a comparative test using independent contrasts. American Journal of Botany 86: 1272–1281.

24. Bond W, Midgley J (1988) Allometry and sexual differences in leaf size. American Naturalist 131: 901–910.

25. Brouat C, Gibernau M, Amsellem L, McKey D (1998) Corner's rules revisited: ontogenetic and interspecific patterns in leaf-stem allometry. New Phytologist 139: 459–470.

26. Gates DM, Schmerl RB (1975) Perspectives of biophysical ecology. Springer.

27. Smith WK (1978) Temperatures of desert plants: another perspective on the adaptability of leaf size. Science 201: 614–616.

28. Karlik JF, McKay AH (2001) Leaf area index, leaf mass density, and allometric relationships derived from harvest of blue oaks in California oak savanna. In: Standiford RB, McCreary DD, Purcell KL (tech. coords.). Proc Fifth Symp Oak Woodlands: Oaks in Californias Changing Landscape, Oct. pp. 22–5.

29. Pretzsch H, Mette T (2008) Linking stand-leval self-thinning allometry to the tree-leval leaf biomass allometry. Trees 22: 611–622.

30. Parkhurst D, Loucks O (1972) Optimal leaf size in relation to environment. The Journal of Ecology 60: 505–537.

31. Beerling DJ, Chaloner WG (1993) The Impact of Atmospheric CO2 and Temperature Changes on Stomatal Density: Observation from Quercus robur Lammas Leaves. Annals of Botany 71: 231–235.

32. Kelly C, Beerling D (1995) Plant life form, stomatal density and taxonomic relatedness: a reanalysis of Salisbury (1927). Functional Ecology 9: 422–431.

33. Abrams MD, Kubiske ME (1990) Leaf structural characteristics of 31 hardwood and conifer tree species in central Wisconsin: influence of light regime and shade-tolerance rank. Forest Ecology and Management 31: 245–253.

34. Körner C, Bannister P, Mark AF (1986) Altitudinal variation in stomatal conductance, nitrogen content and leaf anatomy in different plant life forms in New Zealand. Oecologia 69: 577–588.

35. Poole I, Weyers JDB, Lawson T, Raven JA (2006) Variations in stomatal density and index: Implications for palaeoclimatic reconstructions. Plant, Cell & Environment 19: 705–712.

36. Gindel I (1969) Stomatal number and size as related to soil moisture in tree xerophytes in Israel. Ecology 50: 263–267.

Table 1. Tree trait values, scaling parameters, and other model constants

Parameter	Symbol	Values		Ref.	TRY database		
		Theor.	Empirical		Mean	Median	n
Branching Parameter	n	2	—	[1]	—	—	—
Branching Exponent	a	1	—	[1]	—	—	—
Terminal Branch (Petiole) Radius	r_N	—	0.4 mm	[1]	—	—	—
Terminal Branch (Petiole) Length	l_N	—	4 cm	[1]	—	—	—
Proportionality Constant For Metabolism	β_1	—	0.257 liter day^{-1} cm$^{-\eta_1}$	[4]	—	—	—
Exponent for Metabolism	η_1	2	1.788	[1, 4]	—	—	—
Root to Stem Mass Proportionality	β_3	—	0.423	[3, 6]	—	—	—
Root Absorption Efficiency	γ	1/3	—	this study	—	—	—
Average Leaf Area	a_l	—	13 cm^2	[21–30]	62.10 cm^2	11.76 cm^2	77000
Depth of a Stoma	z_s	—	10 μm	[7]	—	—	—
Leaf Stomatal Density * (averaged over both leaf sides)	δ_s	—	220 stomata mm^{-2}	[31–36]	120 mm^{-2}	101 mm^{-2}	363
Area of a Stoma*	a_s	—	235.1 μm^2 $= \pi\,(17.9/2)^2$	[7, 33, 36]	491 μm^2	459 μm^2	2159
Stomatal Area per Leaf Area* (Stomatal Density × Area of a Stoma)	$\delta_s a_s$	—	.051	[7, 31–36]	.059	.047	—
Leaf Absorptivity (full spectrum)	α	—	0.50	[7, 8]	—	—	—
Soil Reflection Coefficient (full spectrum)	ξ_s	—	0.30	[7, 8]	—	—	—
Deep Canopy Reflection Coefficient (full spectrum)	ξ_c^*	—	0.22	[7, 8]	—	—	—
Proportionality Constant for Canopy Radius	β_5	—	35.24 cm m$^{-\eta_3}$	SI of [11]	—	—	—
Exponent for Canopy Radius	η_3	1	1.14	SI of [11]	—	—	—
Leaf Emmisivity	ϵ	—	0.95	[7, 8]	—	—	—

*It should be noted that our model relies on the bulk property of "Stomatal Area per Leaf Area" for calculations requiring an evaporative flux area a_f. Thus the sub-parameters a_s and δ_s need not agree with the TRY database as long as the bulk property $\delta_s a_s$ does.
